# "Getting Under Your Skin”: First Reported Case of Transient Reactive Phlebitis Involving Vancomycin Infusion Therapy

**DOI:** 10.7759/cureus.18471

**Published:** 2021-10-04

**Authors:** Obteene Azimi-Ghomi, Andrew Napier

**Affiliations:** 1 Department of General Surgery, Kendall Regional Medical Center, Miami, USA; 2 Department of Emergency Medicine, Kendall Regional Medical Center, Miami, USA

**Keywords:** vancomycin, phlebitis, transient reactive phlebitis, vermiform, rash

## Abstract

We discuss a case of a 54-year-old female presenting with uncomplicated diverticulitis failing outpatient oral antibiotic therapy. In the emergency department, the patient was placed on intravenous (IV) vancomycin therapy and developed an atopic, vermiform ascending rash running exclusively along with the venous distributions of the left arm proximal to the vancomycin IV infusion site. We discuss different types of vancomycin-associated cutaneous drug reactions. We also review the literature regarding previous descriptions of this type of drug reaction, known as transient reactive phlebitis (TRP).

## Introduction

Vancomycin is a commonly utilized antibiotic in the hospital setting, primarily as empiric therapy for suspected beta-lactam-resistant Gram-positive bacteria. Numerous adverse drug reactions have been known to occur with vancomycin therapy, cutaneous drug reactions being the most common. These drug reactions are typically due to a localized or systemic drug-mediated histamine release. Transient reactive phlebitis (TRP) is a rarely reported localized drug reaction occurring after infusion of medications. Though its etiology is unknown and its presentation quite dramatic, it is relatively well tolerated and its effects are temporary.

## Case presentation

**Figure 1:**
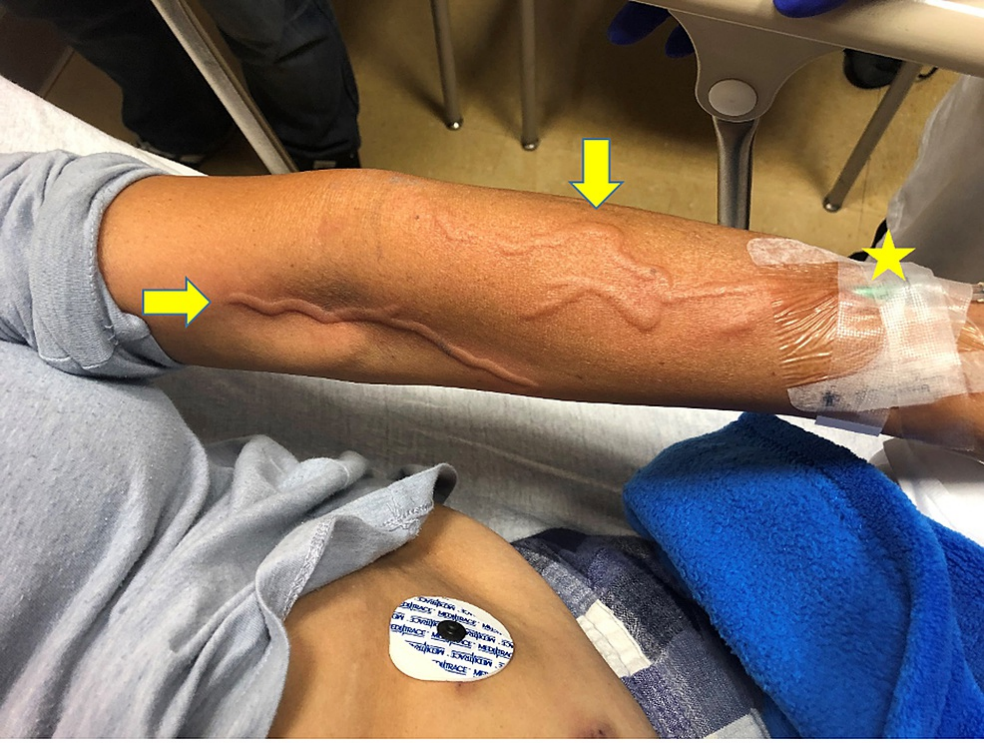
Phlebitis reaction on the patient’s left arm (arrows) in the venous drainage of the vancomycin infusion. The infusing cannula is marked (star).

**Figure 2:**
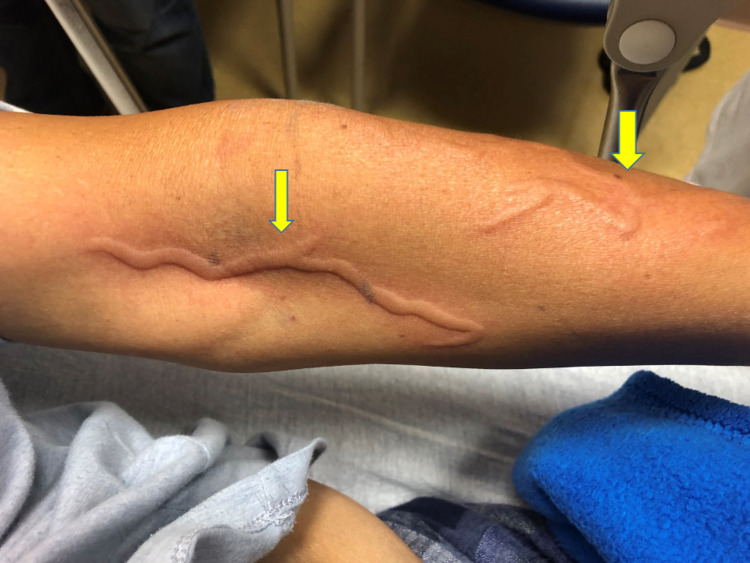
Phlebitis reaction involving the cephalic and median antecubital veins of the left arm (arrows).

A 54-year-old Hispanic female with a history of medication-controlled hypertension presented to our service with fever, chills, and lower abdominal pain. She had been previously seen by our surgical services for her first episode of uncomplicated sigmoid diverticulitis and discharged on an oral antibiotic regimen of metronidazole and levofloxacin. The patient reported that at home her pain progressively worsened, and she returned to seek further care. On presentation, she was afebrile, with a heart rate of 88 beats per minute, and normotensive. Physical examination demonstrated left lower quadrant tenderness to palpation but was otherwise unremarkable. While awaiting computed tomography (CT) scanning, empiric IV antibiotic therapy with vancomycin and piperacillin-tazobactum was initiated by the emergency department physician. Within 30 minutes of vancomycin initiation, the patient began experiencing a vermiform-appearing ascending rash running exclusively along the venous distributions of the left arm proximal to the vancomycin IV infusion site (Figures 1-2). The patient denied fevers, chills, tenderness, or changes in motor or sensory function in the affected extremity. At that time, the patient had not yet received the IV piperacillin-tazobactum.

Laboratories including a complete blood count (CBC) demonstrated a leukocytosis of 14.9K with 82% neutrophilia and the presence of a left shift. The remaining CBC and chemistry panel were within normal limits. Given the appearance of the rash shortly after initiation of IV vancomycin, the patient was presumed to have a TRP. The vancomycin infusion was stopped and the rash resolved approximately 15 minutes after cessation of therapy. The patient was subsequently treated with intravenous piperacillin-tazobactum only, which she appeared to tolerate well. CT scans demonstrated a persistent uncomplicated sigmoid diverticulitis. The patient was continued on piperacillin-tazobactum therapy and experienced an uneventful hospital course with discharge three days later on oral amoxicillin-clavulanate. No further episodes of antibiotic reactions were noted during the patient's hospital course.

## Discussion

Vancomycin is a glycopeptide antibiotic commonly used in the hospital setting for empiric antibiotic therapy as well as anti-methicillin-resistant *Staphylococcus aureus* (anti-MRSA) coverage [1]. Vancomycin has been documented to cause a variety of adverse effects [1,2]. The most common vancomycin-associated drug reaction are cutaneous adverse reactions [2]. Other forms of vancomycin drug reactions include ototoxicity, nephrotoxicity, drug-induced fever, neurological changes, hematological changes, and gastrointestinal symptoms [2].

Vancomycin-associated cutaneous drug reactions are associated with two types of reactions, with both attributed to the direct or indirect mediation of histamine release [2-4]. The first is due to the direct induction of histamine release by mast cells due to the antibiotic molecule and its metabolic products [2]. One well-documented example is “Red-Man Syndrome” which is associated with a rapid rate of vancomycin infusion. The second type of cutaneous drug reaction secondary to vancomycin infusion is a hypersensitivity reaction mediated by immunoglobulin E (IgE) that results in histamine release and has been associated with eosinophilia [2-4]. This type of reaction is seen in up to 5% of patients receiving vancomycin [2].

Vancomycin-associated cutaneous drug reactions encompass up to 47.9% of all vancomycin adverse drug reactions [2,3]. Twenty-nine percent of cutaneous drug reactions attributed to vancomycin occurred only during the administration of the antibiotic [3]. Treatment of these adverse cutaneous reactions is typically supportive, and symptoms usually resolve with cessation of vancomycin therapy [2-4]. One caveat with “Red-Man Syndrome” is that symptoms typically resolve after the IV vancomycin infusion rate is titrated down or stopped completely [2,3].

There have been several other reports of a TRP occurring after initiation of a medication drip, though none have reported vancomycin as a causative agent. Previously reported medications include morphine, pethidine, rocuronium, propofol, ciprofloxacin, diphenhydramine, and eptifibatide [5-13]. TRP is isolated to the recipient extremity and occurs downstream to the infusing intravenous cannula [5,10-13]. Symptoms include a vermiform rash comprising the region of phlebitis and may be associated with mild pain [5-8,10,13]. Pruritis, urticaria, and febrile episodes have not been reported. As the namesake may suggest, symptoms are transient and completely resolve with cessation of the causative medication [8,10,13]. Symptoms resolve within minutes to hours of cessation. Although not well understood, several hypothesized mechanisms have been proposed. These include directly mediated histamine release, activation of the kallikrein-kinin system with bradykinin generation, direct activation of c-nociceptors, and the release of local mediators. [8,10-13]. A histamine-mediation reaction appears less likely to be the cause of TRP due to the absence of typical histamine-associated symptoms such as pruritis or urticaria, as well as the commonly utilized anti-histamine medication diphenhydramine having been reported in TRP [13]. Dilution of the infusing medication, slowing the rate of infusion, or use of a large vein for IV infusion access may also help ameliorate symptoms and reduce the risk of developing TRP in susceptible patients [13]. 

## Conclusions

Transient reactive phlebitis is a rarely reported adverse effect of IV drug infusion. We discuss the first reported case of TRP occurring following IV vancomycin therapy. TRP has also been rarely reported following infusion of other medications, most commonly anesthetics and narcotics. All documented TRP cases, including ours, reported resolution of symptoms following cessation of therapy. Vancomycin-associated cutaneous drug reactions comprise up to 48% of all adverse effects seen with vancomycin, with over half occurring in isolation. These reactions can occur due to direct hypersensitivity caused by vancomycin or its metabolites, or may be an IgE-mediated histamine release seen with type 1 hypersensitivity reactions. We report the first document episode of TRP in vancomycin therapy. This drug reaction is a rare and dramatic yet relatively benign adverse drug effect, with symptoms promptly resolving following cessation of the IV infusion. The pathophysiologic mechanism of TRP is still unknown and further research is required to elucidate its exact cause.
